# Proximal subungual onychomycosis of *digitus minimus* due to *Aspergillus brasiliensis*

**DOI:** 10.11604/pamj.2020.35.79.20762

**Published:** 2020-03-19

**Authors:** Manjula Mehta, Jyoti Sharma, Sonia Bhonchal Bhardwaj

**Affiliations:** 1 Department of Microbiology, Dr Harvansh Singh Judge Institute of Dental Sciences and Hospital, Panjab University, Chandigarh, India

**Keywords:** Onychomycosis, dermatophytes, *Aspergillus brasiliensis*

## Abstract

Onychomycosis is a common nail infection caused by dermatophytes, non-dermatophytic molds (NDMs) and yeast. *Aspergillus spp.* are emerging etiological agents of non-dermatophyte mold onychomycosis (NDMO). Though this is usually of cosmetic concern, it may also cause pain and discomfort to the patient. The toenail is more commonly involved as compared to fingernail. The nails are discoloured and disfigured. Onychomycosis may expose the patient to cellulitis of lower extremities. The clinical presentation of dermatophytic and NDM onychomycosis is more or less similar, which creates problem in the diagnosis. Fingernail infection may cause social and psychological problem to the patient if fingernail is involved. Incidence of onychomycosis has been seen more in immunosuppressed individuals, where it is of more serious medical concern. In the present study we are reporting a case of proximal subungual onychomycosis (PSO) due to *Aspergillus brasiliensis*.

## Introduction

Onychomycosis which is also known as *tinea unguium* [1] is a fungal infection of toenail or fingernail [2], which may involve any component of the nail unit like nail bed, nail matrix or nail plate. It constitutes majority of all the commonly known nail abnormalities [3]. It may cause discomfort, pain and disfigurement, which may also lead to psychological problems. The most common symptoms of onychomycosis are nail discoloration, nail thickening and sometimes separation of nail from nail bed [4]. There are certain predisposing factors to this condition like diabetes mellitus, immunosuppression due to HIV, peripheral arterial disease, etc. [5]. It is caused by a variety of fungi like anthropophilic dermatophytes, NDMs and *Candida* [6, 7]. Out of the etiological agents of onychomycosis, dermatophytic molds are the main cause known so far. Onychomycosis due to NDMs is becoming increasingly prevalent now. Though, they are lesser known to invade the nail plate [8]. Among these NDMs, *Aspergillus* species are increasingly being reported as the causative agent of onychomycosis [9]. As they mimic the clinical picture of onychomycosis due to dermatophytes, the diagnosis becomes difficult and unreliable [10].

## Patient and observation

**Figure 1:**
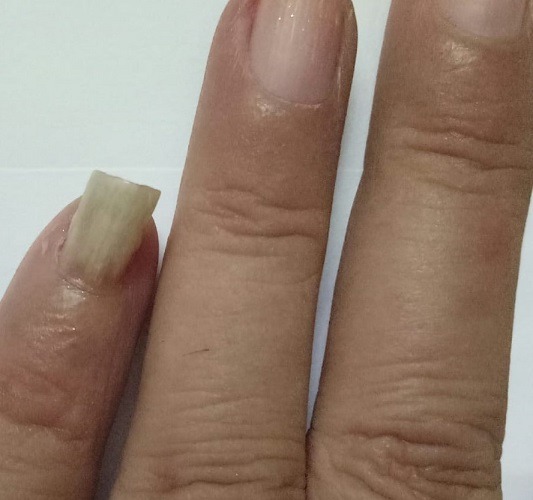
Lesion at the time of presentation

**Figure 2:**
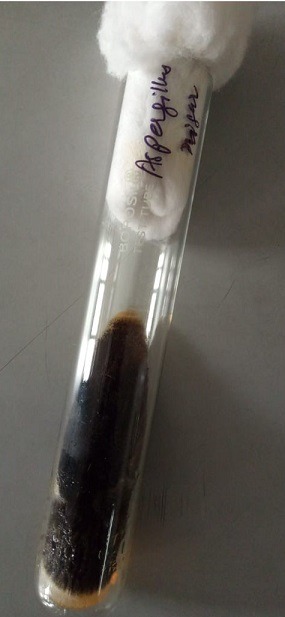
Growth on SDA

**Figure 3:**
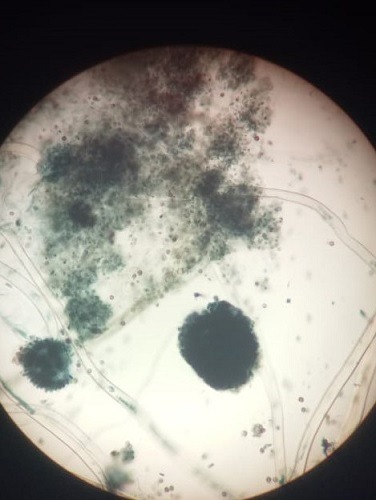
LCB staining showing septate hyphae and biseriate phialides

In this report we describe a case of onychomycosis in a 55-year-old female with no underlying clinical illness. She was presented with a lesion on *digital minimus* nail of her left hand suggestive of proximal subungual onychomycosis (Figure 1). It was also accompanied by paronychia and hyperkeratosis. The nail also showed discoloration which covered the entire nail plate. Nail margins were fragile and broken but the nail plate remained intact. Although the patient had this lesion for months, she reported until it was painful. It was tender too. Patient history revealed that she suffered nail trauma twenty years ago. The patient was non-diabetic and long-term steroid use was also ruled out. Routine blood examination done for blood cell abnormalities, liver function and kidney function test showed normal values. Direct microscopy of the nail specimen was done by using 40% potassium hydroxide (KOH) which revealed the presence of septate hyphae with dichotomous branching. The sample was grown on Sabouraud Dextrose Agar (SDA) with and without cycloheximide and chloramphenicol. Tubes were then incubated at 25°C and 37°C for four weeks. The fungal growth was seen in SDA tube without cycloheximide and chloramphenicol. Initially the growth was whitish which turned black later on. The reverse of the slant was yellowish (Figure 2). Lactophenol cotton blue mount of the growth showed septate hyphae and biseriate phialides which covered the entire vesicle with radiating conidial heads (Figure 3). The culture was repeated thrice at an interval of fourteen days to confirm the findings. All the three cultures showed similar findings. The patient was treated orally with 250mg terbinafine daily and topical amorolfine (5%) nail lacquer twice a week for three months. The patient responded well to the treatment. Mycological culture post treatment did not reveal any fungal elements.

## Discussion

Although dermatophytes and yeast like fungi are more frequently associated as etiological agents of onychomycosis [2], *Aspergillus spp.* is establishing its role as emerging cause of onychomycosis. This is an important laboratory contaminant so its diagnosis is very crucial. Moreover the clinical picture is non-specific and mimics other types of onychomycosis making diagnosis more difficult [10]. The most important prerequisite to establish NDM as the causative agents of onychomycosis are the repeat positive direct microscopy and culture [9]. We need to rule out the presence of dermatophytes as well. In our case also, dermatophytes were ruled out. The dichotomous hyphae were observed on KOH mount and the same findings were reported after the culture was repeated. The most important clinical manifestation of PSO is that, it is accompanied by painful paronychia without pus discharge [2]. The similar picture has been presented in our case.

## Conclusion

Onychomycosis, especially of the fingernail may put a significant impact on social, emotional and occupational life of the patient. Therefore, specific mycological identification of the etiological agent is important to provide appropriate treatment and to formulate strategies for the proper management of the infection.

## Competing interests

The authors declare no competing interests.

## References

[1] Seyyed Amin Ayatollahi Mousavi, Samira Salari, Sanaz Hadizadeh (2015). Evaluation of antifungal effect of silver nanoparticles against Microsporum canis, Trichophyton mentagrophytes and Microsporum gypseum. Iran J Biotechnol.

[2] Biancia Maria Piraccini, Aurora Alessandrini (2015). Onychomycosis: a review. J Fungi.

[3] Gabriela Moreno, Roberto Arenas (2010). Other fungi causing onychomycosis. Clin Dermatol.

[4] (2017). Onychomycosis-Dermatologic Disorders Merck Mannuals Professional Edition.

[5] Richard Scher, Phoebe Rich, David Pariser, Boni Elewski (2013). The epidemiology, etiology and pathophysiology of onychomycosis. Semin Cutan Med Surg.

[6] John Thorne Crissey (1998). Common dermatophyte infections: a simple diagnostic test and current management. Postgrad Med.

[7] Maggi E Kemna, Boni E Elewski (1996). A US epidemiologic survey of superficial fungal diseases. J A, Acad Dermatol.

[8] Malcolm Richardson (1997). Effects of lamisil and azole antifungals in experimental nail infection. Dermatology.

[9] Erick Obed Martinez Herrera, Stefanie Arroyo Camarena, Diana Luz Tejada Garcia, Carlos Franscisco Porras Lopez, Roberto Arenas (2015). Onychomycosis due to opportunistic molds. An Bras Dermatol.

[10] Marjan Motamedi, Zeinab Ghasemi, Mohammad Riza Shidfar, Leila Hosseinpour, Hissein Khodadadih, Kamair Zomorodian K (2016). Growing incidence of non-dermatophyte onychomycosis in Tehran, Iran. Jundishapur J Microbiol.

